# Yellow Fever in New Orleans

**Published:** 1847-09

**Authors:** 


*Yellow fever in New Orleans.*—The Board of Health in New Orleans have officially announced the prevalence of the Yellow Fever in every part of the city of New Orleans, and the unacclimated are warned to absent themselves.

